# Factors Associated with HPV Vaccine Refusal among Young Adult Women after Ten Years of Vaccine Implementation

**DOI:** 10.3390/ijerph15040770

**Published:** 2018-04-17

**Authors:** Vincenzo Restivo, Claudio Costantino, Tiziana Francesca Fazio, Nicolò Casuccio, Claudio D’Angelo, Francesco Vitale, Alessandra Casuccio

**Affiliations:** 1 Department of Science for Health Promotion and Mother Child Care “G. D’Alessandro”, University of Palermo, Via del Vespro 133, 90127 Palermo, Italy; vincenzo.restivo@unipa.it (V.R.); claudio.costantino01@unipa.it (C.C.); fazio.tiziana78@gmail.com (T.F.F.); francesco.vitale@unipa.it (F.V.); 2 Department of Medical Prevention, Local Health Unit of Palermo, Palermo 90141, Italy; trudeau@libero.it (N.C.); claudiodangelo847@alice.it (C.D.)

**Keywords:** human papillomavirus, vaccine refusal, hesitancy, women, school based, Health Belief Model, gynaecologist, general practitioner, survey, catch up

## Abstract

In Italy, the Human Papillomavirus (HPV) vaccination was implemented for twelve years old girls in 2007, but its coverage was lower than the recommended level. Sicily is one of the Italian administrative regions with lower vaccination coverage, with a value of 59% for those born in 1996 increasing to 62% coverage for those born in 1999. The aim of the study was to investigate factors associated with the refusal of HPV vaccination among young adult women of Palermo, Italy. The study was approved by the Ethics Committee of the Policlinico “Paolo Giaccone” Hospital (Palermo 1) and the questionnaire was validated in a convenience sample representing 10% of the young women. A cross-sectional study was conducted through the administration of a telephone questionnaire, consisting of 23 items on HPV infection and vaccination knowledge based on the Health Belief Model framework. The eligible population were young women (18–21 years old) who had at least a vaccination among all included in the Sicilian vaccination schedule, without starting or completing HPV vaccination. Overall, 141 young women were enrolled (response rate 22%). Among them, 84.4% were unvaccinated and 15.6% had at least one dose of the HPV vaccine. In multivariate analysis, the factors associated with the refusal of the HPV vaccination were a bachelor’s as the education level (OR = 10.2, *p* = 0.041), lower participation at school seminar on HPV (OR = 0.2, *p* = 0.047) and lower perception of HPV vaccine benefits (OR = 0.4, *p* = 0.048). Public health educational program focusing and tailored on benefits perception of HPV vaccine and HPV disease severity, carried out at school or during medical visits, can be useful to improve HPV vaccination uptake.

## 1. Introduction

Human Papillomavirus (HPV) vaccination represents the best strategy for primary prevention of cervical cancer. HPV vaccines have high efficacy against cervical pre-cancerous lesions, if given to females before they are exposed to the virus; therefore, the World Health Organization (WHO, Geneva, Switzerland) recommends offering HPV vaccination to pre-adolescent girls [1].

As of January 2018, 30 of 31 European (EU) countries had implemented HPV vaccination. Target age, financing and vaccine delivery differed among countries [2]. In 2014, mean EU coverage accounted for 53% in the primary target, and organised catch-up. Even if a lower number of primary cohorts were invited, African countries reported a mean 88% HPV vaccination coverage [3].

In Italy, HPV vaccination is free and has been actively offered to all girls during their 12th year of life since 2007, and the National Health Department established a target vaccination coverage of 95% within five years of the start of the campaign [4,5]. However, despite several promotional activities, vaccination coverage is largely unsatisfactory, ranging from 27% to 83% among administrative regions [6]. Sicily is one of the Italian administrative regions with lowest HPV immunization coverage, with a value of 59.5%, 58.7% and 62.1% for full HPV vaccination in the 1997, 1998, and 1999 birth cohorts, respectively [6]. Consistent differences were reported between subjects who received at least one dose of HPV vaccine and who complete the full vaccination schedule. In Sicily, HPV vaccine coverage for at least one dose were 70.3%, 63.4% and 69.2% in 1997, 1998, and 1999 birth cohorts, respectively [6].

Previous studies conducted in Italy and in Europe among adolescent and young women have indicated that common reasons for not receiving the HPV vaccine were the perception of low risk or not needing the vaccine, lack of vaccine awareness, doubt about the safety and efficacy of the vaccine, fear of side effects, inadequate testing of novel vaccines that may be harmful and weaken the immune system, lack of physician recommendations and cost of the vaccine [7,8,9].

The Health Belief Model (HBM) attempts to explain and predict health behaviours, and is used in assessing health-behaviour interventions by focusing on the attitudes and beliefs of individuals. The HBM has been used extensively to study vaccination beliefs and behaviours, and has also been used in vaccination research to identify people’s perceptions of disease and vaccination [7,10,11].

The objective of the study was to investigate factors associated with refusal of HPV vaccination using HBM among young adult women of the City of Palermo, Italy, after ten years of vaccine implementation.

## 2. Materials and Methods

An observational study was conducted among young adult women of the local health unit (LHU) of Palermo (Italy), including girls born from 1996 to 1999 (overall 1996–1999 Palermo birth cohorts *n* = 26,153). The sample was recruited through the vaccination registries of two public vaccination services of Palermo LHU. These services were selected to be as representative as possible of the Palermo geographical area, relative to knowledge, attitudes and behaviours towards HPV vaccination and reliable sources of information. The vaccination registry was filled in for each girl residing in the area of public vaccination services, and who had performed at least one immunization recommended by the Sicilian vaccination schedule. A structured questionnaire was administered through the telephone by a trained healthcare professional from May to September 2017. The eligible population was represented by young adult women who refused or did not expect complete HPV vaccination schedule. The interviewer invited eligible young women to participate in the study, ringing up them to explain objectives of the study and data treatment. At a later time, the interviewer rang young women to administer the questionnaire. The exclusion criteria from the study were: erroneous telephone numbers, not responding after at least six attempts, already vaccinated, and refusal to participate in the study. At the beginning of the interview, informed consent was obtained and survey aims were explained as well as methods used to ensure confidentiality of data. The study was approved by the Ethics Committee of the Policlinico “Paolo Giaccone” Hospital (Palermo 1) on 5 April 2017 (protocol number 04/2017). The questionnaire consists of 23 items based on previous studies conducted about HPV infection knowledge in Sicily and HBM in Europe, and it was divided into two sections (8–9). The first concerned demographic characteristic, HPV infection knowledge and use of gynaecologist services. The second section consisted of HPV vaccination knowledge and health belief investigating: perceived susceptibility of risk of developing cervical cancer (one item), perceived severity of the disease and its consequences (one item), perceived benefits related to vaccination (four items), perceived benefits related to HPV vaccination (three items), perceived vaccination barriers (two items) and perceived HPV vaccination barriers (three items) using HBM as the theoretical framework. The available response options, using a five point Likert scale, were: 1 = absolutely disagree; 2 = disagree; 3 = neither disagree nor agree; 4 = agree; and 5 = absolutely agree. In order to make the results easier to understand, all questions were scored towards beliefs that would result in adherence to vaccination. Therefore, lower scores reflect stronger beliefs about vaccination refusal. This questionnaire was validated in a convenience sample representing approximately 10% of the young women.

Statistical analyses were performed using STATA v14.2 software (StataCorp LLC, College Station, TX, USA). For all analyses, *p*-value ≤ 0.05 was assumed to indicate significance (two-tailed). Normal distribution and homogeneity of variables were tested with Kolmogorov–Smirnov and Levene’s test, respectively. Mean values with standard deviation (SD) or median with interquartile range(IQR) were calculated for quantitative variables, while frequencies were counted for qualitative variables. Mean data were compared by a one-way analysis of variance (ANOVA) with Scheffe’s post hoc test, while comparisons of differences in the medians were analysed with the Mann–Whitney test. A univariate logistic regression analysis was performed in order to evaluate the factors associated with refusal of HPV vaccination. Study covariates, which were found to be significantly associated with the study outcome after the univariate analysis (*p* < 0.1), were evaluated into the multiple logistic regression models. Multivariate analysis was performed to investigate the independent effect of a risk or protective factor after adjustment for one or several other factors or to adjust for confounding variables. Only age was considered an a priori confounder.

Furthermore, a subset was selected to conduct an exploratory factor analysis on reasons to refuse HPV vaccination. Principal factor extraction was used followed by orthogonal rotation, which allows correlation among the factors. A three factor model was fitted, examining factor loadings for each model.

## 3. Results

**Figure 1:**
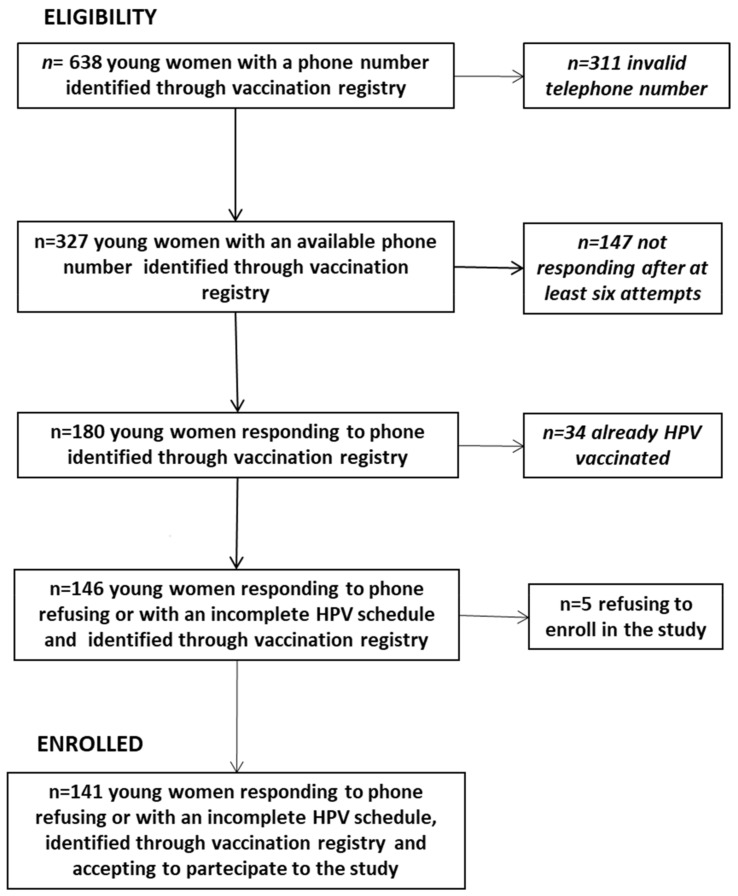
Flow chart for exclusion from the study.

Overall, 638 young women were eligible, although 72% of them did not have a useful phone number (erroneous phone number or not responding). Figure 1 describes reasons for exclusion from the study. Main reasons for exclusion from the study were: to have an invalid telephone number (49%, *n* = 311) or not being available after at least six attempts (23%, *n* = 147). Only 5% (*n* = 34) of women had already performed HPV vaccination outside Sicily.

A total of 141 young women were enrolled in the study. Of these, 84.4% (*n* = 119) were unvaccinated and 15.6% (*n* = 22) received at least one dose of the HPV vaccine. Demographic characteristics, HPV infection knowledge and use of gynaecologist services of young adult women were described in Table 1. The median age was 19 years (IQR = 18–20) and the enrolled women more frequently had a high school diploma (66.7%) followed by those with bachelor degrees (26.2%). Furthermore, 31.2% (*n* = 44) of young women participated in school informative meetings about HPV, 58.2% (*n* = 82) had a complete sexual intercourse, 11.3% (*n* = 16) a sexually transmitted disease (STD) and 50.3% (*n* = 71) a gynaecologist visit. Young women refusing vaccination more frequently had a school diploma (odds ratio (OR) = 4.56, *p* = 0.034) than women with an incomplete HPV vaccine schedule. On the other hand, young women refusing vaccination had less commonly taken part in a school informative meeting about HPV (OR = 0.38, *p* = 0.043), and they had a lower amount of gynaecologist visits (OR = 0.24, *p* = 0.009).

As showed in Table 2, main sources of information about HPV vaccination were paediatrician/general practitioners (42.5%, *n* = 60), followed by gynaecologists (33.3%, *n* = 47) and parents (24.8%, *n* = 35). The HBM answers on benefits of all vaccines had a mean score of 2.5 (SD = 0.1), benefits of HPV vaccine 2.5 (SD = 0.1), barriers of all vaccines 2.6 (SD = 0.1), barriers of HPV vaccine 2.0 (SD = 0.1), susceptibility of disease 4.3 (SD = 0.1) and disease severity 3.2 (SD = 0.1). Young women refusing vaccination had lower scores on HBM questions of perceived HPV vaccination benefits (OR = 0.42, *p* = 0.002), perceived HPV vaccination barriers (OR = 0.46, *p* = 0.008) and perceived severity (OR = 0.50, *p* = 0.022), compared to women with at least one vaccination dose received.

In multivariate analysis (Table 3), factors associated with the failure to perform HPV vaccination compared to performing at least one dose were bachelor’s as the educational level (OR = 10.62, *p* = 0.028), the lower participation in school seminars on HPV (OR = 0.25, *p* = 0.028) and the lower perception of anti-HPV vaccine benefits (OR = 0.41, *p* = 0.044).

Main reasons for vaccination refusal of HPV vaccine were lack of information (39.5% *n* = 47), followed by fear of vaccine adverse events (33.6% *n* = 40), vaccine was not efficacy (15.1% *n* = 18) and logistic reasons (11.8% *n* = 14).

An exploratory factor analysis using the scree test and factor rotation, three factors were isolated as demonstrated in Table 4. Factor analysis showed that perceived benefits related to HPV vaccination and age had higher factor loading (0.85 and 0.64) for lack of information explaining 56% of total variance. Furthermore, perceived benefits related to HPV vaccination, perceived vaccination barriers and education were highest factor loading for both logistic reason (0.83, 0.63, 0.70 respectively) and fear of vaccine adverse events (0.87, 0.71, 0.54 respectively) explaining 57% and 55% of total variance, respectively. Furthermore, perceived benefits related to HPV vaccination (0.84) and perceived vaccination barriers (0.71) were the highest factor loading to believe that vaccine was not efficacy and explain 57% of total variance.

## 4. Discussion

The Sicily Health Authority offered universal HPV vaccinations to all 10-year-old to 12-year-old girls. However, there were notable variations in vaccination coverage among Sicilian LHUs and, in more detail, lower vaccination coverage was reported in the LHU of Palermo. The present study investigated reasons for refusal of the HPV vaccine among young adult women of the City of Palermo, and explored their perceptions and attitudes using the HBM.

One of the main factors associated with HPV vaccine refusal was a higher education level. This finding was already presented in several studies, but it has a discordant trend. In England, Jan et al. showed that LHUs with more educational deprivation had higher rates of vaccination for all doses. In this study, educational deprivation includes individuals with no qualification or the lowest levels of qualification [12]. On the other hand, in Greece, Michail et al. observed that female students who studied at university were more likely to be vaccinated than female students who attended a Technological Institute [13]. In our setting, it was possible that young women with a higher educational level were more sceptical about trusting or accepting information about vaccination at face value. It is also possible that women with the lowest educational level had higher levels of health literacy, as observed by Lee et al. In this study, young adults with lower educational level had better HPV literacy and higher rates of both HPV vaccination initiation and completion, signalling the importance of increasing education and knowledge about HPV to the public [14].

Another predictor associated with HPV vaccine refusal was not taking part in school informative meetings about HPV. In Italy, the HPV vaccination offer was carried out outside the school setting and therefore the organization of meetings on the HPV was carried out sporadically. Unlike Italy, the UK provides the HPV vaccine in school (offered to all girls aged 12). The UK HPV vaccination programme had several variations: some schools hold assemblies to promote and discuss the programme in advance of vaccination days, and other schools offer science lessons associated with HPV vaccination [11]. In Sweden, according to National law, all first year upper secondary school students (aged 16 years) are offered a health interview with the school nurse, who provides a dialogue regarding several preventive topics such as sexual health and relationships [15]. In Italy, school health service was introduced in 1968 and concerned preventive and emergency medicine [16]. In 2004, this law was abrogated because it referred to an epidemiological, social, scholastic context extremely modified in its features [17]. Furthermore, school health service was also superseded by the same legislation about the organization of the Italian health service by paediatricians [17]. On the other side, the ‘Valore project’ showed that Italian pre-adolescents were interested in acquiring additional information about HPV vaccination, and identified school as a setting where they are free to express themselves without fear of being judged, especially if the dialogue takes place with trusted teachers [18]. These results suggested the possible key role of schools in the promotion of correct information about HPV vaccination. Additionally, to achieve a higher uptake of the vaccine, it could be offered in the context of school-based voluntary vaccination and information campaigns, as already implemented in other countries [15,19]. It has been showed that acceptance rate of the HPV vaccine is considerably lower (19–71%) in the lack of school-based programs than in the presence of the programs (65–86%) [20].

In addition, participants with a lower score for perceptions about HPV vaccinations’ benefits were more likely to be unvaccinated. This finding is in agreement with Marlow et al. where participants with a low score for HPV vaccination benefits (safety and efficacy of HPV vaccination) were more likely to be unvaccinated [7]. A possible explanation would be that a threatened individual did not expect to accept the recommended health action unless it was perceived as feasible and efficacious. While acceptance of personal susceptibility to a condition also believed to be serious was held to produce a force leading to behaviour, the particular course of action to be taken depends upon beliefs regarding the effectiveness of the various actions available in reducing the disease threat [11]. Evaluation of the perceptions about benefits, barriers, severity and susceptibility of HPV vaccines could play a key role in the development of targeted educational campaigns that would increase the intention to get the HPV vaccine [12,21]. Public Health professionals should provide tailored information to reinforce strength of recommendations regarding HPV vaccination, especially with parents of young women [21,22].

A consistent lack of information is mainly reported among young women who never performed vaccination. This data was similar to what was reported in a study conducted in the USA and in a survey conducted in Italy [8,23]. In another study conducted in the Netherlands, lack of information can be interpreted as being in a modifiable phase for HPV vaccine acceptance, given the clear lack of vaccination preference and prevailing emotions regarding the topic. Those with this perspective are not likely to vaccinate against HPV, as they feel no sense of urgency in terms of perceived threat or benefit [24]. The provisions of unambiguous information about benefits of vaccination and risks of the disease, clarifying also doubts, fears, and risk of severe side-effects, were fundamental communicative strategies in influencing awareness of young women of HPV vaccination [4].

Young women who reported fear of vaccine adverse events as the reason to refuse HPV vaccination were suffering from comorbidities and were critical of the roles of the pharmaceutical industry in HPV vaccination. Furthermore, their inclination to refuse HPV vaccination was dominated by fear of potential long-term side effects, in accordance with other findings [8,24]. Those young women were unlikely to accept HPV vaccination, as the perceived threat was minimal and the perceived barriers clearly outweighed any benefit. Cues to action indicate a need for more updated and adequate information.

Finally, another reason for refusing HPV vaccination was revealed to be logistical reasons. The most frequently reported components of this category were the distance from the vaccination center and the lack of time to administer HPV vaccines. In particular, the latter was reported above all in a study as a reason for not completing the vaccination schedule [22]. According to the factor analysis, a better knowledge of the benefits of HPV vaccination may increase the adhesion even in those reporting organizational problems. Furthermore, improved accessibility to public vaccination services, for example by opening on weekends, could also increase vaccination coverage.

There are two main limitations of this study. Firstly, the study was based on self-reported information, so personal perceptions may have been overestimated, although self-reporting was recognized as a cost-effective and feasible method for gathering data from large population samples. Secondly, the study had a low participation rate; therefore, the possibility of non-response bias should be taken into consideration. The low participation rate was mainly due to invalid phone numbers in the vaccination registry, suggesting a reorganization of the telephone catch-up procedure of Palermo vaccination services.

## 5. Conclusions

These findings suggest that educational interventions focused on sexual transmitted diseases and conducted in a school setting may be necessary to enhance HPV vaccination rates among Sicilian girls. Therefore, supplying correct and unambiguous information to young women about vaccine efficacy and safety, and the value of vaccinations in preventing cervical cancer may be needed in order to increase HPV vaccination coverage in the future.

## Figures and Tables

**Table 1:** Demographic characteristics, HPV infection knowledge, and use of gynaecologist services of young adult women.

Variables	Total (*n*= 141)	Incomplete Vaccination (*n* = 22)	Refusal Vaccination (*n* = 119)	Crude OR	95% CI	*p*
Age, mean (SD)	19 (18–20)	19 (18–20)	19 (18–20)	0.80	0.55–1.15	0.266
Education, *n* (%)						
Secondary school	10 (7.1)	4 (18.2)	6 (5.0)	1		
High school	94 (66.7)	12 (54.4)	82 (68.9)	4.56	1.12–18.52	0.034
University	37 (26.2)	6 (27.4)	31 (26.1)	3.44	0.74–16.03	0.155
Family members, median number (IQR)	4 (3–5)	4 (3–5)	4 (3–5)	0.85	0.47–1.52	0.582
Smoking habit, *n* (%)						
No	119 (84.4)	6 (27.4)	103 (86.6)	1		
Yes	22 (15.6)	16 (72.6)	16 (13.4)	0.41	0.14–1.21	0.108
Drinking habit, *n* (%)						
No	117 (83.0)	4 (18.2)	99 (83.2)	1		
Yes	24 (17.0)	18 (81.8)	20 (16.8)	0.91	0.28–2.97	0.875
Papillomavirus knowledge, *n* (%)						
No	9 (6.4)	1 (4.5)	8 (6.7)	1		
Yes	132 (93.6)	21 (95.5)	111 (93.3)	0.66	0.08–5.56	0.703
HPV can cause genital warts, *n* (%)						
No	114 (80.9)	17 (77.3)	97 (81.5)	1		
Yes	27 (19.1)	5 (22.7)	22 (18.5)	0.77	0.26–2.31	0.643
HPV can cause cervical cancer, *n* (%)						
No	13 (9.2)	1 (4.5)	12 (10.1)	1		
Yes	128 (90.8)	21 (95.5)	107 (89.9)	0.42	0.05–3.44	0.422
I don’t know diseases caused by HPV, *n* (%)						
No	129 (91.5)	21 (95.5)	108 (91.8)	1		
Yes	12 (8.5)	1 (4.5)	11 (9.2)	2.14	0.26–17.46	0.478
Taking part in a school informative meeting about HPV, *n* (%)						
No	97 (68.8)	11 (50.0)	86 (72.3)	1		
Yes	44 (31.2)	11 (50.0)	33 (27.7)	0.38	0.16–0.97	0.043
Current relationship status, *n* (%)						
Stable relationship	80 (56.7)	14 (63.6)	66 (46.8)	1		
Non-stable relationship	4 (2.8)	2 (9.0)	2 (1.7)	0.21	0.03–1.63	0.137
Single	57 (40.5)	6 (27.4)	51 (51.5)	1.80	0.65–5.02	0.259
Being sexually active, *n* (%)						
No	59 (41.8)		53 (44.5)	1		
Yes	82 (58.2)		66 (55.5)	0.47	0.17–1.28	0.138
Suffering of STDs’, *n* (%)						
No	125 (88.7)	6 (27.4)	108 (91.8)	1		
Yes	16 (11.3)	16 (73.6)	11 (9.2)	0.35	0.11–1.12	0.077
To perform gynaecologist visit, *n* (%)						
No	70 (49.7)	5 (22.7)	65 (54.6)	1		
Yes	71 (50.3)	17 (77.3)	54 (45.4)	0.24	0.08–0.70	0.009
PAP test knowledge, *n* (%)						
No	18 (12.8)	2 (9.3)	16 (13.4)	1		
Yes	123 (87.2)	20 (90.7)	103 (86.6)	0.64	0.14–3.02	0.577
To have a PAP test, *n* (%)						
No	112 (79.4)	15 (68.2)	97 (81.5)	1		
Yes	29 (20.6)	7 (31.8)	22 (18.5)	0.49	0.18–1.33	0.161

STDs: sexual transmitted diseases. PAP test: Papanicolau test. HPV: Human Papillomavirus. SD: standard deviation. IQR: interquartile range.

**Table 2:** HPV vaccination knowledge and HBM (Health Belief Model) questions of young adult women.

Variables	Total (n = 141)	Incomplete Vaccination (*n* = 22)	Refusal Vaccination (*n* = 119)	Crude OR	95% CI	*p*
Informative source about anti-HPV vaccination						
Gynaecologist, *n* (%)						
No	94 (66.7)	13 (59.1)	81 (68.1)	1		
Yes	47 (33.3)	9 (40.9)	38 (31.9)	0.68	0.26–1.72	0.414
Public vaccination services, *n* (%)						
No	137 (97.2)	21 (95.5)	116 (77.5)	1		
Yes	4 (2.8)	1 (4.5)	3 (2.5)	0.54	0.05–5.47	0.605
Parents, *n* (%)						
No	106 (75.2)	19 (86.4)	87 (73.1)	1		
Yes	35 (24.8)	3 (13.6)	32 (26.9)	2.33	0.64–8.41	0.197
Paediatrician/General Pratictioner, *n* (%)						
No	81 (57.5)	13 (59.1)	68 (48.5)	1		
Yes	60 (42.5)	9 (40.9)	51 (51.5)	1.08	0.43–2.73	0.865
Social network, *n* (%)						
No	135 (95.8)	21 (95.5)	114 (95.8)	1		
Yes	6 (4.2)	1 (4.5)	5 (4.2)	0.92	0.10–8.29	0.942
Perceived vaccination benefit I 1–4, mean (SD)	2.5 (0.1)	2.6 (0.1)	2.5 (0.1)	0.76	0.34–1.68	0.494
Perceived HPV vaccination benefit I 5–7, mean (SD)	2.5 (0.1)	3.1 (0.1)	2.4 (0.1)	0.42	0.23–0.75	0.002
Perceived vaccination barrier I 8–10, mean (SD)	2.6 (0.1)	2.9 (0.1)	2.6 (0.1)	0.49	0.23–1.04	0.063
Perceived HPV vaccination barrier I 11–12, mean (SD)	2.0 (0.1)	2.4 (0.2)	1.9 (0.1)	0.46	0.26–0.81	0.008
Perceived susceptibility I 13, mean (SD)	4.3 (0.1)	4.5 (0.2)	4.2 (0.1)	0.73	0.42–1.26	0.262
Perceived severity I 14, mean (SD)	3.2 (0.1)	3.6 (0.2)	3.1 (0.1)	0.50	0.28–0.91	0.022

I-1 I do not trust in vaccinations; I-2 vaccinations are not effective and do not prevent diseases; I-3 It is not necessary to receive all vaccines; I-4 It is preferable to get the disease and to be protected naturally than to vaccinate; I-5 I do not consider safe the HPV vaccine; I-6 I believe that if I receive the HPV vaccine, I will not be protected from cervical cancer; I-7 I believe that if I receive the HPV vaccine, I will not be protected against HPV infection; I-8 I do not have enough information about infection prevented by vaccines; I-9 I do not have enough information about vaccines; I-10 The vaccination unit is hard to access; I-11 Paediatrician or general practice discouraged me from getting the HPV vaccine; I-12 Social media or the internet discouraged me from getting the HPV vaccine; I-13 My sexual behaviour is safe; I-14 I do not believe that HPV is exceptionally harmful.

**Table 3:** Multivariate logistic regression of factor associated with refusal of HPV vaccine.

Covariates	Adjusted OR	95% CI	*p*
Age	0.69	0.43–1.12	0.139
Education			
Secondary school	1		
High school	5.33	0.83–34.35	0.078
University	10.62	1.29–87.52	0.028
Taking part in a school informative meeting about HPV			
No	1		
Yes	0.25	0.07–0.93	0.028
To perform gynaecologist visit			
No			
Yes	0.59	0.17–2.09	0.414
Suffering of STDs			
No	1		
Yes	0.59	0.17–2.09	0.414
Perceived HPV vaccination benefit I5–7	0.41	0.17–0.98	0.044
Perceived vaccination barrier I8–10	0.83	0.29–2.35	0.731
Perceived HPV vaccination barrier I11–12	0.69	0.31–1.56	0.375
Perceived severity I14	0.80	0.31–2.00	0.635

STDs = sexual transmitted diseases. I-5 I do not consider safe the HPV vaccine; I-6 I believe that if I receive the HPV vaccine, I will not be protected from cervical cancer; I-7 I believe that if I receive the HPV vaccine, I will not be protected against HPV infection; I-8 I do not have enough information about infection prevented by vaccines; I-9 I do not have enough information about vaccines; I-10 The vaccination unit is hard to access; I-11 Paediatrician or general practice discouraged me from getting the HPV vaccine; I-12 Social media or the internet discouraged me from getting the HPV vaccine; I-13 My sexual behaviour is safe; I-14 I do not believe that HPV is exceptionally harmful.

**Table 4:** Factor analysis of reasons to refusal HPV vaccine.

	Reasons to Refusal HPV Vaccine											
	Lack of Information			Logistic Reasons			Vaccine Was Not Efficacy			Fear of Vaccine Adverse Events		
Variables	Factor 1	Factor 2	Factor 3	Factor 1	Factor 2	Factor 3	Factor 1	Factor 2	Factor 3	Factor 1	Factor 2	Factor 3
Education	0.1617	0.6080	0.1896	0.3430	0.6995 *	−0.3473	0.1836	0.4921	0.3058	0.1420	0.3136	0.5424 *
Age	−0.0626	0.6364 *	0.0409	−0.0387	0.6472	0.0199	−0.0921	0.6535	0.0436	−0.0940	0.6990	0.0723
Perceived benefits related to vaccination	0.7121	−0.0493	−0.1266	0.8036	−0.0331	−0.0682	0.7943	−0.1137	0.1669	0.7423	−0.1650	0.1398
Perceived benefits related to HPV vaccination	0.8541 *	−0.0922	−0.1248	0.8341*	−0.1496	0.2301	0.8392 *	−0.0830	−0.2299	0.8661*	−0.1106	−0.0275
Perceived vaccination barriers	0.1832	0.6208	−0.1410	−0.0044	0.4906	0.6271 *	0.1319	0.7137 *	−0.2381	0.1965	0.7135 *	0.0622
Perceived HPV vaccination barriers	0.7297	0.1739	0.1338	0.5406	0.0557	0.4597	0.5548	0.2705	−0.4969	0.6718	0.1731	0.0458
Perceived susceptibility of risk of developing cervical cancer	−0.2648	0.6271	−0.1579	−0.2129	0.6180	0.1541	−0.1271	0.5439	0.3933	−0.1834	0.4093	0.5386
Perceived severity of the disease and its consequences	0.5502	0.1123	−0.5667	0.5427	0.0413	0.3963	0.6934	0.1383	0.0242	0.6501	0.2928	−0.2461

* values indicate the highest loading weights.

## References

[1] World Health Organization (2009). WHO position paper: Human papillomavirus vaccines. Wkly. Epidemiol. Rec..

[2] European Centre for Disease Prevention and Control Vaccine Schedule. http://vaccine-schedule.ecdc.europa.eu/Pages/Scheduler.aspx.

[3] Bruni L., Diaz M., Barrionuevo-Rosas L., Herrero R., Bray F., Bosch F.X., de Sanjosé S., Castellsagué X. (2016). Global estimates of human papillomavirus vaccination coverage by region and income level: A pooled analysis. Lancet Glob. Health.

[4] Giambi C., D’Ancona F., Del Manso M., De Mei B., Giovannelli I., Cattaneo C., Possenti V., Declich S. (2014). Local Representatives for VALORE. Exploring reasons for non-vaccination against human papillomavirus in Italy. BMC Infect. Dis..

[5] Italian Health Minister Piano Nazionale Prevenzione Vaccinale PNPV 2017–2019. http://www.salute.gov.it/imgs/C_17_pubblicazioni_2571_allegato.pdf.

[6] Italian Health Minister Papillomavirus (Hpv), i dati sulle coperture vaccinali aggiornati al 2015. http://www.salute.gov.it/portale/news/p3_2_1_1_1.jsp?lingua=italiano&menu=notizie&p=dalministero&id=2828.

[7] Marlow L.A.V., Waller J., Evans R.E.C., Wardle J. (2009). Predictors of interest in HPV vaccination: A study of British adolescents. Vaccine.

[8] Firenze A., Marsala M.G., Bonanno V., Maranto M., Ferrara C., Giovannelli L., Restivo V. (2015). Facilitators and barriers HPV unvaccinated girls after 5 years of program implementation. Hum. Vaccines Immunother..

[9] Donadiki E.M., Jiménez-García R., Hernández-Barrera V., Sourtzi P., Carrasco-Garrido P., López de Andrés A., Jimenez-Trujillo I., Velonakis E.G. (2014). Health Belief Model applied to non-compliance with HPV vaccine among female university students. Public Health.

[10] Montgomery K., Smith-Glasgow M.E. (2012). Human papillomavirus and cervical cancer knowledge, health beliefs, and preventive practices in 2 age cohorts: A comparison study. Gend. Med..

[11] Janz N.K., Becker M.H. (1984). The Health Belief Model: A decade later. Health Educ. Q..

[12] Jean S., Elshafei M., Buttenheim A. (2018). Social determinants of community-level human papillomavirus vaccination coverage in a school-based vaccination programme. Sex. Transm. Infect..

[13] Michail G., Smaili M., Vozikis A., Jelastopulu E., Adonakis G., Poulas K. (2014). Female students receiving post-secondary education in Greece: The results of a collaborative human papillomavirus knowledge survey. Public Health.

[14] Lee H.Y., Lee J., Henning-Smith C., Choi J. (2017). HPV literacy and its link to initiation and completion of HPV vaccine among young adults in Minnesota. Public Health.

[15] Grandahl M., Rosenblad A., Stenhammar C., Tydén T., Westerling R., Larsson M., Oscarsson M., Andrae B., Dalianis T., Nevéus T. (2016). School-based intervention for the prevention of HPV among adolescents: A cluster randomised controlled study. BMJ Open.

[16] Presidential Decree of Italian Republic 22 Dicembre 1967, n. 1518 Regolamento per L’applicazione del Titolo III del Decreto del Presidente della Repubblica 11 Febbraio 1961, n. 264, Relativo ai Servizi di Medicina Scolastica. http://www.gazzettaufficiale.it/eli/id/1968/06/06/067U1518/sg;jsessi.

[17] Italian Health Minister Documento Finale del Gruppo di Lavoro per la Semplificazione Delle Procedure Relativamente Alle Autorizzazioni, Certificazioni ed Idoneita’ Sanitarie. http://www.epicentro.iss.it/discussioni/obsolete/pdf/Documento%20EBP%20finale.pdf.

[18] Giambi C., Del Manso M., De Mei B., D’Ancona F., Giovannelli I., Cattaneo C., VALORE Working Group (2013). VALORE Project: Local and Regional Assessment of Vaccination Campaigns against HPV: Promote Conscious Adherence to Vaccination.

[19] Skinner S.R., Cooper Robbins S.C. (2010). Voluntary schoolbased human papillomavirus vaccination: An efficient and acceptable model for achieving high vaccine coverage in adolescents. J. Adolesc. Health.

[20] Kessels S.J., Marshall H.S., Watson M., Braunack-Mayer A.J., Reuzel R., Tooher R.L. (2012). Factors associated with HPV vaccine uptake in teenage girls: A systematic review. Vaccine.

[21] Palmeri S., Costantino C., D’Angelo C., Casuccio N., Ventura G., Vitale F., Pojero F., Casuccio A. (2017). HPV vaccine hesitancy among parents of female adolescents: A pre-post interventional study. Public Health.

[22] Liddon N.C., Hood J.E., Leichliter J.S. (2012). Intent to receive HPV vaccine and reasons for not vaccinating among unvaccinated adolescent and young women: Findings from the 2006–2008 National Survey of Family Growth. Vaccine.

[23] Zimet G.D., Weiss T.W., Rosenthal S.L., Good M.B., Vichnin M.D. (2010). Reasons for non-vaccination against HPV and future vaccination intentions among 19–26 year-old women. BMC Womens Health.

[24] Patty N.J.S., van Dijk H.M., Wallenburg I., Bal R., Helmerhorst T.J.M., van Exel J., Cramm J.M. (2017). To vaccinate or not to vaccinate? Perspectives on HPV vaccination among girls, boys, and parents in the Netherlands: A Q-methodological study. BMC Public Health.

